# Dysplastic Nevus or Melanoma? A Commentary on the “Patient and Doctor Anecdotal Observations” Article

**DOI:** 10.1111/ijd.17772

**Published:** 2025-04-08

**Authors:** Giulia Briatico, Giuseppe Argenziano

**Affiliations:** ^1^ Dermatology Unit University of Campania Naples Italy

**Keywords:** digital imaging, melanoma, nevi, pigmented lesions, skin cancer

1

In their research paper published in the February 2025 issue of the *International Journal of Dermatology*, Liu et al. [1] describe the case of a patient with a positive familial and personal history of melanoma. The patient underwent treatment for 5 years in a high‐risk skin cancer clinic. Three months after the first visit, the patient's general practitioner recommended a punch biopsy of a pigmented lesion, which was diagnosed histopathologically as melanoma in situ associated with a junctional dysplastic nevus. Subsequent wide local excision, which incorporated three adjacent pigmented lesions, led to a second in situ melanoma diagnosis. The patient was then monitored every 3 months for 5 years using 3D total‐body photography, and no further excisions were recommended. This story is paradigmatic of the possible overdiagnosis of melanoma in situ in patients with multiple melanocytic nevi. In essence, melanocytic nevi in these patients could be atypical not only clinically and dermoscopically but also histopathologically, leading to the possible overdiagnosis of melanoma in situ in lesions that would not have progressed if eventually left untreated.

However, this is only speculation since no studies have demonstrated that non‐progressing/stable lesions might be histopathologically diagnosed as melanoma. A study should be carried out to prove the overdiagnosis bias, including excised lesions that have proven stable over time. Indeed, melanoma is a changing lesion, at times faster, other times slower [2]. In contrast, lesions that are stable over time provide de facto reassurance of their biological benign nature. Consequently, if stable lesions were histopathologically diagnosed as melanoma, this could provide evidence that the issue of overdiagnosis does exist, especially in the context of patients with multiple atypical nevi.

Digital monitoring is a crucial procedure for patients with multiple atypical nevi. This technique guarantees that only changing lesions are excised while stable lesions, albeit atypical, are followed up. This strategy, therefore, maximizes melanoma detection while minimizing the unnecessary excision of benign lesions [3].

In the context of digital monitoring, some potential issues should be addressed. First, it is necessary to correctly select the lesions that warrant monitoring at the baseline examination. Patients must be examined carefully, considering their age, phototype, and signature nevus pattern. A comparative approach is required in order to find equivocal lesions that should be excised immediately. Nodular lesions with equivocal features should be excised [3] as well. Further examinations could be scheduled after 3–6 or 6–12 months based on specific criteria, including the total nevus count and the history of previous melanoma. Both short‐term and long‐term monitoring are helpful in detecting melanoma [4]. However, in our experience, only a minority of melanomas are detected after a three‐month follow‐up. Quite often, melanoma in situ grows slowly, and sometimes, 1–2 years are needed to detect such slow‐growing melanomas [2].

Another critical point is that the side‐by‐side comparison of lesions during follow‐up examinations is an operator‐dependent technique. Sometimes, it is difficult to assess the relevance of slight changes occurring in a given lesion, and confocal microscopy can assist in these cases. Artificial intelligence approaches are underway and will eventually be ready to be applied as a support tool in real life. Lastly, high‐risk patients need not only expert dermatologists but also expert dermatopathologists to evaluate their lesions. The differential diagnosis between dysplastic nevus and melanoma in situ is still a diagnostic challenge [5] that could be overcome by a more widespread use of second opinions with improved cooperation among expert dermatopathologists.

In conclusion, digital monitoring is a difficult procedure that requires the experience of a dedicated team to reduce the possibility of bias and improve diagnostic accuracy. Liu et al.'s clinical problem calls for improved collaboration between clinicians and pathologists that may lead to a better classification of difficult‐to‐diagnose melanocytic proliferations.

## Conflicts of Interest

The authors declare no conflicts of interest.
